# QbD Approach in Cosmetic Cleansers Research: The Development of a Moisturizing Cleansing Foam Focusing on Thickener, Surfactants, and Polyols Content

**DOI:** 10.3390/gels10080484

**Published:** 2024-07-23

**Authors:** Cătălina Bogdan, Diana Antonia Safta, Sonia Iurian, Dyana Roxana Petrușcă, Mirela-Liliana Moldovan

**Affiliations:** 1 Department of Dermopharmacy and Cosmetics, Faculty of Pharmacy, “Iuliu Hațieganu” University of Medicine and Pharmacy, 12 I. Creangă St., 400010 Cluj-Napoca, Romania; catalina.bogdan@umfcluj.ro (C.B.); dyana.roxa.petrusca@elearn.umfcluj.ro (D.R.P.); 2 Department of Pharmaceutical Technology and Biopharmacy, Faculty of Pharmacy, “Iuliu Hațieganu” University of Medicine and Pharmacy, 41 V. Babeș St., 400012 Cluj-Napoca, Romania; sonia.iurian@umfcluj.ro

**Keywords:** cleansing foams, DoE, dry skin hygiene, mild surfactants, xanthan gum, polyols

## Abstract

Cleansing products, particularly innovative cosmetic foams, must efficiently remove impurities with minimal impact on the skin barrier and have a favorable sensory profile. The choice of product ingredients is crucial to ensure the optimal characteristics. The current study aims to provide a comprehensive framework for understanding the variability in the characteristics of a cleansing foam to achieve desired properties. The novelty of this study lies in the combination of ingredients for their potential synergistic and complementary effects in cleansing dry skin, as well as the application of Quality by Design (QbD) elements to develop and optimize the formulation of cleansing foam. The effects of varying the concentration of mild surfactants, polyols, and gel-forming agents on the properties of the gels and of the generated foams were studied. Significant influences of the formulation factors were observed: an increased ratio of xanthan gum positively impacted the texture properties of the gel, whereas higher concentrations of surfactants had a negative impact on these parameters. Additionally, increasing the polyols ratio was found to negatively influence the foaming property and stability of the foam. The study established an optimal formulation of a cleansing foam with a ratio of 0.45% xanthan gum, 26.19% surfactants and 2.16% polyols to be used for dry skin hygiene.

## 1. Introduction

The performance requirements of cleansing products encompass efficient removal of environmental pollutants, make-up, sebum, and microorganisms from the skin’s surface [1]. The cleansing action is primarily attributed to surfactants or “surface acting agents”, amphiphilic compounds used to clean skin through chemical or physical mechanisms. Surfactants clean the skin’s surface by emulsifying the lipids, which are further easily washed, while physical cleansing involves the mechanical removal of the impurities from the surface of the skin. Surfactants should have a minimal impact on the barrier function of the skin [2]. Particularly for dry skin, it is crucial to use cleansers with minimal defatting activity and a neutral to slightly acidic pH to avoid further damage [3].

Progress in surfactant technology led to the formulation of affordable and performant cleansers. Among the high variety of cleansing products, cosmetic foams represent an innovative category for skin hygiene, with various advantages such as ease of application and effective and fast removal of both hydrophilic and lipophilic impurities. According to European Pharmacopoeia’s definition, foams are obtained at the moment of administration by dispersing a significant volume of gas in a liquid [4]. Generally, foam-based delivery systems contain the active ingredients and the dispersed solid or liquid components that become gaseous by pressing the valve of the conditioning bottle, due to the contained surfactants. Foams can be obtained from non-aerosol recipients, as well as by releasing the ready-to-use product upon pump activation [5]. The easy application and good spreadability of foams lead to increased acceptability and consumer satisfaction [1,2,5].

The main disadvantage of the foams is the fact that they are thermodynamically unstable, so formulation studies are needed to ensure foam stability. Moreover, their composition must be well chosen to guarantee efficiency and sensorial properties. To fully understand various formulation components and processes affecting the final product, a Quality by Design (QbD) approach is therefore very important, helping to achieve a quality-based development [1,5].

One of the main challenges of the cosmetic industry is to ensure consistent product quality during the manufacturing process, given its importance for maintaining safety, efficacy, and regulatory compliance, while also achieving cost-effective production. Therefore, the adaptation of QbD principles into cosmetic development plays a crucial role in producing high-quality products that meet safety requirements and fulfill user expectations. Moreover, the integration of QbD elements yields additional environmental advantages, since the industry is increasingly focused on sustainability, aiming to reduce energy and material usage and generate less waste in the production of cosmetics.

The development of cosmetic foams within the QbD framework considers the specific characteristics of these formulations and accelerates the development process. The development of a product through QbD involves the profiling step with the selection of a quality target product profile (QTPP) and determining the Critical Quality Attributes (CQAs) [1]. Appearance and bubble size, viscosity of the liquid formulation, foamability, foam density, consistency and texture, or drug content and drug release for medicated foams are just a few of the features with important for foam quality [1,6]. Further, the identification of risk factors is conducted to establish potential risk variables both in the early stages of development and throughout the development process, as additional knowledge about the product is obtained. In line with the QbD principles, Falusi et al. (2022) identified and categorized the CQAs with the highest potential impact in the quality profile, followed by determining the material attributes and the process parameters that exert the highest risk on the CQAs. The type and ratio of polymers, as well as the stirring speed and time, were deemed as the most impactful factors on properties like foam volume stability, foam expansion, or cross point [5].

However, the QbD approach usually involves the use of Design of Experiments (DoE) as a risk mitigation tool, which can lead to a Design Space described as a multidimensional combination and interaction of input variables shown to guarantee the product’s quality [7,8]. The development of cosmetic products using DoE has as an advantage the ability to quantify the relationships between the risky variables and the properties of the product, allowing the prediction of the experimental conditions that lead to the desired characteristics [9].

Therefore, the current study aims to provide a framework to assess and understand the variability of the characteristics of a cleansing foam containing mild surfactants and suitable excipients to ensure adequate properties for dry skin hygiene. The factors influencing its quality attributes were analyzed through DoE leading to the development of an optimal formulation. The novelty of this study lies in the combination of ingredients chosen for their potential synergistic and complementary effects in cleansing dry skin, and in the application of the QbD approach to set the optimum factors for an improvement in product performance. Moreover, this study is one of the few in the literature that use QbD elements to develop a cleansing foam, studying both the gel and generated foam characteristics.

## 2. Results and Discussion

A thorough understanding of the interactions between surfactants and the compromised stratum corneum barrier is necessary for the formulation of cleaning products for dry skin. Due to their many advantages over conventional skincare products, foams are becoming more attractive. They are also considered a user-friendly solution. The product is delivered in an exact amount thanks to the dispenser pump, which also lowers the risk of microbial contamination.

To develop the foam, mild surfactants like lauryl glucoside, decyl glucoside, and sodium cocoyl isethionate were chosen due to their excellent cleansing efficacy and skin tolerance. Lauryl glucoside and decyl glucoside are nonionic surfactants with good foaming properties and soft caring after feeling [6]. They do not produce a significant decrease in skin moisture [10]. Sodium cocoyl isethionate is an anionic surfactant with high foaming power [11] and produces rich creamy foam with a moisturizing and soothing feeling [6,12].

Foams are characterized by a low density and are influenced significantly by surface tension gradient. Three steps characterize the foaming process: initiation and formation, stability, drainage and rupture. Initially, foam formation is due to large gas voids which create a high liquid-gas interface. Surfactant solutions stabilize this interface, creating a surface tension gradient that prevents liquid drainage and favors the persistence of the foam. While the foam formation is influenced by the dynamic surface pressure, foam stability is influenced by the surface dilatational rheology. Thus, low concentrations of surfactants create elastic monolayers that prevent foam formation and higher concentrations yield a viscoelastic surface necessary for the stability of the foams. Initially, during the foam formation, the foam is predominantly liquid, afterward the foam coarsens as bubble size increases and lamellar distances decrease. Increasing the viscosity of the interlamellar liquid by adding water-soluble polymers limits the film drainage [13].

The active ingredients: sodium PCA (pyrrolidone carboxylic acid), *Aloe vera* oil, *Cedrus atlantica* bark oil, *Cupressus sempervirens* oil and *Copaifera officinalis* resin oil were chosen for their interesting properties to restructure the barrier and enhance the appearance of dry skin. The selected ingredients may act synergistically and complementary to gently clean the skin and provide the hydration needed for dry skin.

*Aloe vera* L. (*Asphodelaceae* family) is considered one of the most potent, commercially important, and popular plants in the research field of skin products. Different parts of the plant contain about 200 active phytocompounds, namely amino acids, polysaccharides, enzymes, vitamins, minerals, saponins, anthraquinones, lignin, and salicylic acid [14]. In addition to providing a protective barrier for the skin and retaining moisture, *Aloe vera* may promote the healing of skin damage which may be associated with dry skin [14,15].

*Cedrus atlantica* (Endl.) Manetti ex Carriere (*Pinaceae* family) contains several bioactive compounds, including oxygenated sesquiterpenes and sesquiterpene hydrocarbons. The major compounds encountered are α-pinene, himachalene, β-himachalene, cis-α-atlantone, himachalol, germacrene D, and β-copaene [16,17] *Cedrus atlantica* oil exhibits high antibacterial and antifungal activity, remarkable antioxidant activity, good anti-inflammatory, and important depigmenting activity through tyrosinase inhibition [16].

*Copaifera officinalis (Fabaceae* family) contains volatile compounds like sesquiterpenes e.g., β-caryophyllene, α-copaene, β-bisabolene, trans-α-bergamotene with anti-inflammatory, antioxidant, and antimicrobial actions, being used traditionally in the treatment of wounds [18,19].

*Cupressus sempervirens* L. (*Cupressaceae* family) contains volatile oils such as α-pinene, δ-3-carene, limonene, and α-terpinolene that possess antioxidant, antimicrobial, and antibiofilm properties [20,21].

As regards the impact of oil inclusion on foaming systems, the stability of the foams can be reduced by changing the surface tension and increasing liquid drainage [22].

In addition to herbal extracts and vegetal oils, sodium PCA was added to lessen the dryness usually associated with the washing procedure. Sodium PCA, a salt of pyrrolidone carboxylic acid), is naturally present in the stratum corneum as a component of the natural moisturizing factor (NMF). There are many reports of topical PCA applications being effective in treating symptoms of dry skin [23,24]. Furthermore, glycerin, the most well-known humectant, and moisturizer, can keep the skin smooth and thus help to preserve hydration in the top layers of the skin [25]. Xylitol has also been documented for its skin-hydrating effect [26]. As concluded by Korponyai et al., using a combination of glycerol and xylitol can be a useful approach for treating dry skin, by increasing the skin hydration, the quantity of filaggrin, the interdigitation index, decreasing the TEWL (transepidermal water loss) and improving the biomechanical properties of the skin [26]. Moreover, as demonstrated by Szel et al., xylitol can exert anti-irritant and anti-inflammatory effects even at a lower concentration compared to glycerol [27]. In a study conducted by Sagitani et al., the effect of polyols on the formation of the lamellar gel phase for the stability of cream soaps was investigated. The results showed that glycerol was more advantageous than other polyols, such as 1,3-butylene glycol, PEG 400, or dipropylene glycol, in obtaining stable cream soaps [28].

Xanthan gum is a thickening natural-derived agent, that is widely used in cosmetic formulations due to its beneficial properties. It is highly compatible with anionic, amphoteric, and nonionic surfactants and stabilizes foams against separation [11,29]. The addition of different polymers such as xanthan gum, alginates, or cellulose derivatives in foam preparations can be used to enhance the stability of the foams, through the formation of a surfactant-polymer complex [22].

Lathering products are generally more popular among users, but they are not typically recommended for dry skin [30]. However, by combining the appropriate level of surfactants that ensure effective cleansing without damaging skin barrier lipids, along with humectants and plant extracts with antioxidant and anti-inflammatory properties, a balanced product that mitigates the negative effects commonly associated with lathering products can be obtained.

Recently, Falusi et al. developed foams using the QbD concept, studying the influence of different polymers on the quality of the foams. They also noted the good properties of xanthan gum in foam development, specifying that the type and concentrations of different polymers can impact the properties of the product in different ways [5]. Despite the significant contributions of this research, the literature remains scarce on this topic, this article being the only one found on foam development using the QbD approach. Thus, to the best of our knowledge, our article is one of the few publications to explore QbD in the formulation of cleansing foams, underscoring the novelty and importance of our findings. In addition to the research of Falusi et al., the current study aimed to investigate some other factors affecting both the gel and the foam formed, such as surfactants and polyol concentrations, contributing to providing a comprehensive framework for enhancing product quality.

### 2.1. QbD Approach

#### 2.1.1. Definition of QTPP, CQAs

QbD involves identifying and understanding the critical quality attributes (CQAs) of the cleansing foam from the elements that compose the QTPP (Table 1). By defining these early in the development process, manufacturers can ensure the final product meets the desired specifications and performance standards. As outlined in Table 1, the key attributes of successful cleansing products include the following characteristics: easy to spread with appropriate foaming properties, pleasant feeling during the application, non-greasy feeling, residue-free, and additional moisturizing properties while cleaning [30].

#### 2.1.2. Risk Analysis

In Figure S1 and Table S1 (Supplementary Materials), the risk assessment and management of foaming-gels development are highlighted. The potential risks were identified, and the control methods were established. By addressing these risks proactively, the likelihood of product failure or defects is significantly reduced. The factors that had the greatest RPN were the most important in assuring the quality of the final product [1]. Thus, the concentrations of surfactants, polyols, and the gel-forming agent, were further chosen as variation factors in the experimental plan.

#### 2.1.3. Experimental Design

The results obtained after preparing and analyzing the formulations are presented in Table 2.

As the design of experiments selected for this study was a D-Optimal screening design, the mathematical model was able to quantify the independent effects and the second-order interactions. The theoretical model applied to each of the responses is presented below:

Y = const + a1X1 + a2X2 + a3X3 + a4X1X2 + a5X1X3 + a6X2X3

where Const—constant, intercept, a—regression equation coefficients that show the effect of each factor, Y is the response, X1-X3 represent the individual effects, while X1X2, X1X3, and X2X3 are the interactions effects, which show the variation of the response when two factors are changed simultaneously.

Table S2 (Supplementary Materials) presents the revised quantitative factor effects and the associated *p*-values.

#### 2.1.4. Statistical Analysis

For each of the fifteen responses, mathematical models were fitted. According to Table 3, the results obtained show an appropriate fitting of the experimental data with the chosen models. R^2^, the percentage of the variation of the response explained by the model, indicates a good fit, with values above 0.55. The values of Q^2^, defined as the percent of the variation of the response predicted by the model, were higher than 0.45. Consequently, the high values of these two parameters for most response factors indicate a good model. The difference between the two values should be smaller than 0.2–0.3, as smaller differences indicate an appropriately selected model. When the model validity values are higher than 0.25, there is no lack of fit of the model (the model error is in the same range as the pure error) and the values are considered acceptable. The model validity parameter indicates whether the appropriate model type has been chosen. In this case, the validity was good for all answers. The reproducibility, a measure of the systematic error, reflects the variation of the response under identical conditions (pure error), often at the center points, compared to the total variation of the response and it reached values above 0.35. The equations used by the software to calculate the validity and reproducibility are presented below. Table 3 presents the statistical parameters for the ANOVA test and the quality of fit. Excepting Y10, the *p*-value was below 0.05, which indicates a statistically significant model [7,9,31,32].

Validity = 1 + 0.57647 × log10(*p*),

(available only when replicated experiments have been performed).

Reproducibility is a measure of the systematic error, calculated using the following equation [32]:

Reproducibility = 1 − (MSPure error/MSTotal corrected)

#### 2.1.5. The Influence of the Formulation Factors on the Physical Characteristics of the Cleansing Foams

**Figure 1:**
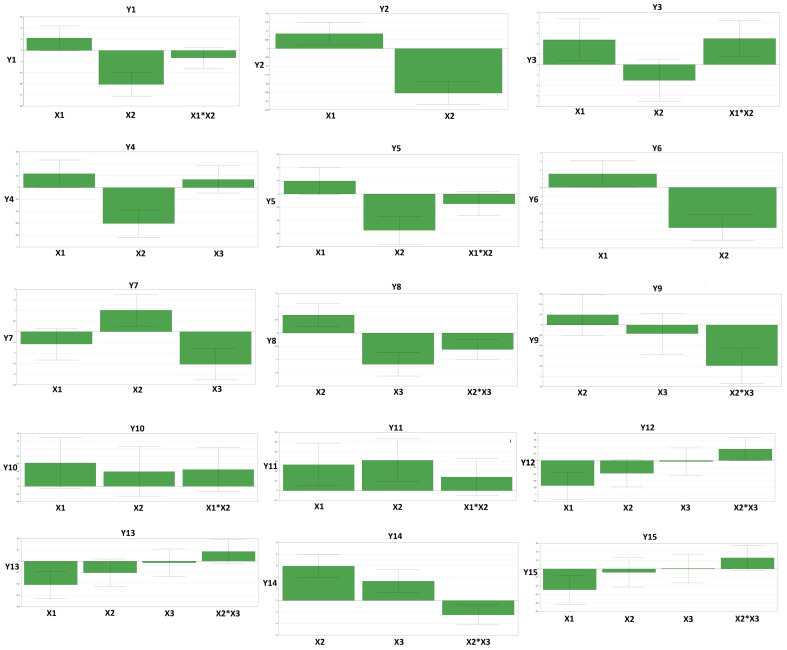
Scaled and centered histograms. Quantitative variables and their variation levels: X1—% xanthan gum, X2—% surfactants (lauryl glucoside, decyl glucoside, sodium cocoyl isethionate), X3—% polyols (glycerin: xylitol); Characterization of the gel: Y1—firmness (g), Y2—consistency (mJ), Y3—stringiness length (mm), Y4–stringiness work done (mJ), Y5—adhesive force (g), Y6—adhesiveness (mJ), Characterization of the foam: Y7—foaming property (mL), Y8—foam stability (mL), Y9—dirt dispersion, Y10—firmness (g), Y11—consistency (mJ), Y12—adhesive force (g), Y13—adhesiveness (mJ), Y14—stringiness length (mm), Y15—stringiness work done (mJ).

The scaled and centered histograms showing the mathematical model coefficients that further indicate the influence of the formulation factors on the parameters of the gels and foams are presented in Figure 1. To a better understanding of the interactions and nonlinear effects, contour plots were also included in Figure S2 (Supplementary Materials). The significant influences are discussed further.

Xanthan gum was selected as an input variable to observe the influences on the physical characteristics of the final products because it is well-known that viscosity-enhancing agents modify the foaming properties in surfactant-based formulations [11]. By increasing the xanthan gum ratio (X1), a positive influence was observed on the values of gel parameters: firmness (Y1), consistency (Y2), stringiness length (Y3), stringiness work done (Y4), and adhesiveness (Y6). These influences can be explained by the thickening and gel network-forming properties of the xanthan gum, which lead to the forming of a more viscous, stickier gel, with an increase in the difficulty in stretching and deforming [29]. Regarding the foam characteristics, an increase in the xanthan gum ratio had also a positive influence on foam consistency (Y11), but a negative influence on the adhesive force (Y12), adhesiveness (Y13), and stringiness work done (Y15). The greater consistency of the foam given by the xanthan gum can be explained by its thickening properties, as for the gel [29]. Inversely as for the gel, xanthan gum decreased the adhesive properties of the foam, probably due to some interactions between the air bubbles and the gel network. Moreover, as the foam network was modified, this led to a decrease in the work needed to stretch the foam.

Increased concentrations of foaming agents and higher viscosities were observed to enhance foam stabilization [22]. Thus, favorable rheological properties and stable foam obtention were observed in the study conducted by Falusi et al., when xanthan gum was used in the preparation of a dermal foam [5].

Generally, self-foaming compositions contain surfactant amounts ranging from 10% to about 35% [33]. In our study, we used a percentage between 15% and 30%, which falls in the previously mentioned interval. A wide experimental range was chosen to observe the impact of extreme surfactant ratios on foam properties. The use of higher ratios of surfactants (X2) determined a negative influence on the values of gel parameters: firmness (Y1), consistency (Y2), stringiness work done (Y4), adhesive force (Y5), and adhesiveness (Y6). These effects can be attributed to the surfactants’ amphiphilic nature, which disrupts the gel network structure. Furthermore, by lowering the surface tension between the substrate and the gel matrix [34], surfactants might lessen the adhesiveness. Higher ratios of surfactants provided higher foaming properties and foam stability and positively influenced the consistency and stringiness length of the foams. As expected, due to the lowering of the surface tension of the liquids and absorbing the air-liquid interface, the surfactants improved foam quality [34]. Subsequently, a high ratio of surfactants had a better ability to stabilize the foam and led to a stronger and more interconnected air bubbles network and a higher consistency and resistance to deformation of the foam. Possible effects on the skin barrier induced by the high ratio of surfactants are reduced through a limited contact time between the surfactant and the skin, as the foam is already formed. Moreover, the selected surfactants are recognized for their mild and skin-friendly properties. Previous studies have shown that mild cleansers do not significantly change TEWL, stratum corneum hydration, or skin surface pH when using such products [30].

Together with the surfactants, the humectants represent the primary ingredients of aqueous-based cleaners. A high percentage of polyols (X3) led to an increase in the values of the stringiness length of the foam (Y14) and a decrease in the values of the foaming property (Y7) and stability of the foam (Y8). The positive effects in stringiness length could be the result of the humectant and plasticizing effect of the polyols, which led to the increase in the hydration level and flexibility of the product and thus to a more stretchable foam [35]. The negative effects on the foam can be explained by the fact that the polyols can increase the viscosity and stickiness of the product, increasing the difficulty of the bubbles forming and maintaining their structure. Additionally, polyols—as hydrophilic molecules—may compete with surfactants and water molecules at the air-liquid interface and inhibit the foam formation or disrupt the foam network [36].

The simultaneous increase in the xanthan gum and surfactant ratios (X1*X2) led to an increase in the stringiness length of the gel (Y3). As previously discussed, the network structure formed by xanthan gum became more robust, while the surfactants facilitated better dispersion and interaction of the ingredients, leading to a more cohesive and elastic texture of the gel. Regarding the simultaneous increase of the surfactants and polyols ratios (X2*X3), their above-explained disruption of the foam network caused by polyols induced a negative influence on the foam stability (Y8), dirt dispersion (Y9) and stringiness length of the foam (Y14).

#### 2.1.6. The pH Determination

The final pH range should be considered when selecting raw materials. For our products, the pH values ranged between 6.4 and 6.8 ensuring good skin compatibility and tolerance. Respecting the skin’s pH is important, especially for cleansing products for dry skin, because of their frequent use and their aggressive potential on sensitive skin. The use of cleansing products at neutral pH is preferred [12]. High pH values of skincare products negatively impact processes like desquamation, the skin's ability to maintain the normal microbiome, and lipid synthesis, essential for maintaining skin health and integrity [37].

#### 2.1.7. Optimization Process

Optimization was based on the previously developed mathematical responses included as CQAs. It aimed at obtaining a suitable product that fulfills the characteristics of the previously established QTPP and was performed using Modde software. Optimization involved applying a set of constraints on the selected responses/CQAs to get the desired quality features. Thus, the consistency and adhesive properties of the gel were minimized, to facilitate the release from the container and to avoid the sticky feeling of the product. Moreover, the foaming property and the stability of the foam were maximized, to achieve a high-quality foam. All these characteristics were chosen to obtain increased cleansing effectiveness and a favorable sensory profile to guarantee the users’ acceptability, given the intended use for dry skin cleansing. The optimal formulation was generated, and then prepared and characterized. The theoretical values predicted, and the practical results obtained after preparation and analysis are close, revealing the good choice of the proposed model (Table 4).

In conclusion, the main advantages of the optimal formulation are emphasized by the following characteristics:

(1) The innovative formulation concept, this study being among the limited research using the QbD approach to develop a cleansing foam.
(2) The content of mild surfactants in optimized concentration ensures effective cleansing while minimizing potential irritation and ensuring suitability for dry skin.
(3) The optimized ratio of polyols increases skin hydration and contributes to the overall efficacy of the cleansing foam.
(4) The optimized ratio of xanthan gum contributes to a better stability of the foam.
(5) The vegetal oils that provide additional moisturizing effects make the product particularly beneficial for dry skin.
(6) Foam for delivery of cosmetic actives: the optimized formulation produces a rich, stable foam and it is designed to clean the skin easily and quickly, ensuring efficient cleansing with minimal contact time between surfactants and skin, thereby reducing the risk of skin dryness or irritation.

The developed cleansing foam is designed to clean the facial skin, carefully avoiding the eye area as it is not intended for the periocular region. For optimal results, it should be used twice daily and gently applied in circular motions to wet skin. The product is thoroughly rinsed with warm water and a moisturizing cream for dry skin should be applied afterward.

#### 2.1.8. Characterization of the Optimal Formulation

pH measurement

The pH of the optimal formulation was 6.58 ± 0.01, a premise to ensure good skin tolerance. The difference in the chemical structure of the synthetic surfactants when compared to traditional soaps, and the pH value of the final product below 7 contributes to the milder nature of the cleansing product [5].

Viscosity measurement

Viscosity significantly affects the rheological behavior of cosmetic cleansers, making its measurement important in product development. The optimal formulation had a viscosity of 71.5 ± 2.29 cPs, a low value necessary for foam generation when using a pump-type dispenser; thicker products risk blocking the dispenser cap and failing to discharge the product properly. Our results were lower than those recorded for other cleansing products (i.e., facial gels or shampoos) due to the presence of thickeners such as gums or cellulose derivates in lower concentrations [38,41]. To ensure the easy release of foam upon activation of the pump, the conditioned product needs to maintain appropriate viscosity.

Regarding the correlation between viscosity and foaming ability, Sheng et al. found that higher viscosities resulted in reduced foamability. They also observed that an increased concentration of xanthan gum increased the viscosity, which led to a decrease in foam height [29].

Foam structure

**Figure 2:**
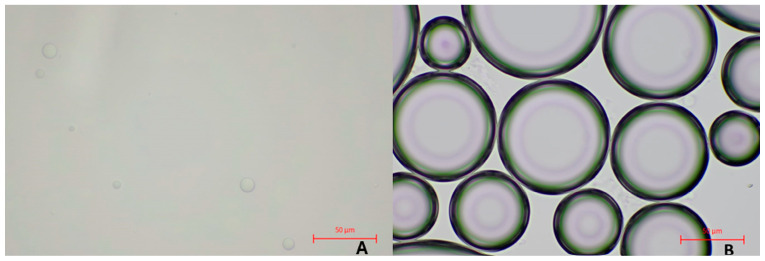
Microscopical image of the gel (**A**) and foam (**B**) at 40× magnification (scale bar 50 μm).

The microscopic image of the optimal formulation of the gel (Figure 2A) displayed a dense, interconnected network of polymer strands forming a three-dimensional matrix. The gel structure was homogenous, and well-formed, with no visible conglomerates or precipitates, assuring the homogeneity of the further foam. The presence of aggregated particles can affect foaming ability, making it essential to ensure the product is homogeneous [12].

The microscopic image of the optimal formulation of foam (Figure 2B) revealed a network of closely packed homogenous bubbles with spherical shapes. The foam structure was uniform and stable, with thin films separating the bubbles. The overall texture was consistent, indicating a well-formed and stable foam matrix. The size distribution was good, slightly varying from approximately 50 µm or below. The obtention of small bubbles was desired to ensure good stability of the foam. To preserve the foam structure and lessen water drainage, smaller foam bubbles raise the air-water contact, enabling the membrane to store significantly more water.

When compared to pressurized aerosol foams, the foam quality generated by foam dispensers that mix air and liquid to create foam differs significantly from that produced by propellant-based methods. Aerosol foams are finer-pored and more viscous than those generated without a gas propellant [22].

Actuation force

**Figure 3:**
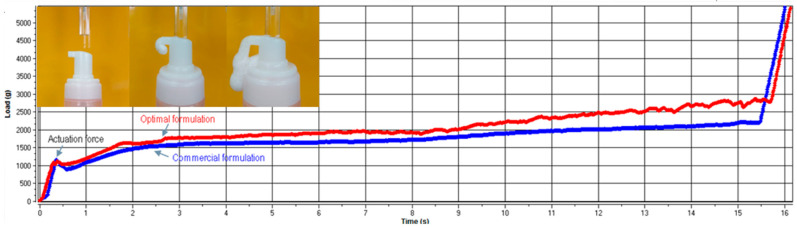
Actuation force measurements for the optimal formulation and a commercial product.

The actuation force is represented by the force necessary to release the foam from the pump-head dispenser [30]. The results of this imitative test depend on different features such as consistency and viscosity of the conditioned gel, influencing the generation of the foam and posing challenges from the manufacturing standpoint. The actuation force vs. time graph shows comparative profiles of the optimal formulation and a commercial foam (Figure 3). As observed, both products exhibit similar profiles, indicating comparable behavior.

## 3. Conclusions

Cleansing products technology is evolving rapidly due to increasing consumer demand for more interesting texture profiles and environmentally sustainable products. In recent years, foams have gained attention due to their several advantages over traditional cleansers. The ease of use, effective cleaning properties, and silky texture make them popular among consumers. However, stability issues and the risk of skin dryness due to the surfactant content pose formulation challenges. Nevertheless, the amount of surfactants is critical for foam formation, making the selection of the appropriate amount essential. Other ingredients such as thickeners and polyols, also impact foam generation and stability. The current study focused on the development of a cosmetic cleansing foam by implementing a systematic DoE as a proof of concept to demonstrate the feasibility of cosmetic development within the QbD framework. The ratios of xanthan gum, surfactants, and polyols were investigated as the most influential factors for the foams. Eleven formulations were obtained by careful choice of the ingredients and the physicochemical properties of both gels and the generated foams were evaluated. All ingredients were selected to be effective and beneficial for the cleansing of dry skin. Their effects on the quality characteristics of the gel and foam were investigated, revealing significant influences of the formulation factors on product characteristics. An optimal formulation of cleansing foam containing 0.45% xanthan gum, 26.19% surfactants, and 2.16% polyols, with suitable characteristics for dry skin, was obtained. These findings provide valuable insights into the formulation of effective cleansing foams for dry skin, highlighting the critical balance between ingredient ratios to achieve optimal product performance. Future studies should assess more formulation factors, such as the ratio of oils or other emollients and different process parameters, to provide a more complete picture of this product category. Additionally, in vitro tests carried out in cell cultures, in vivo performance, skin dryness, and sensory attributes should be explored in subsequent research.

## 4. Materials and Methods

### 4.1. Materials

*Aloe vera* oil, *Cedrus atlantica* bark oil, *Cupressus sempervirens* oil, *Copaifera officinalis* resin oil, lauryl glucoside, decyl glucoside, sodium cocoyl isethionate, glycerin, xylitol, xanthan gum, sodium PCA, and Cosgard (INCI: Benzyl Alcohol, Salicylic Acid, Glycerin, Sorbic Acid), were purchased from Ellemental, Oradea, Romania. PEG-40 hydrogenated castor oil was purchased from Clariant, Muttenz, Switzerland.

### 4.2. Preparation of the Cleansing Foams

The initial step of the process involved the dispersing of xanthan gum in glycerol. Separately, the aqueous phase was prepared by solubilization and gentle mixing of lauryl glucoside, decyl glucoside, sodium cocoyl isethionate, xylitol, and sodium PCA in hot distilled water (70 °C ± 2 °C) on the water bath (BAC-1, Raypa, Barcelona, Spain). The aqueous phase was added to previously obtained xanthan gel and mixed gently until a homogeneous gel was obtained. The ingredients of the oily phase, comprised of *Aloe vera* oil, *Cedrus atlantica* bark oil, *Cupressus sempervirens* oil, *Copaifera officinalis* resin oil, and PEG-40 hydrogenated castor oil, were mixed. Then, the oily phase was added under continuous manual stirring in the aqueous phase previously cooled at room temperature. In the last step, the preservative system was added, and the gel was manually homogenized and conditioned in a foam container. All the formulations, as well as the optimal formulation, were prepared using the same preparation method.

### 4.3. Characterization of the Gels and Foams

To evaluate the quality of foaming cleansing products, several characteristics are commonly tested: texture parameters, foaming property, foam stability, foam structure, viscosity, and pH.

#### 4.3.1. Characterization of the Gels

The texture analysis of both the gel and the generated foam was assessed using the CT3 Texture Analyzer (Brookfield Engineering Laboratories, Middleboro, MA, USA). The accessories of the texture analyzer were chosen based on the sample type and the test performed, considering the very distinct characteristics of gels and foams. Thus, the TA-DEC probe and TA-BT-KIT fixture are generally used for testing semisolids, such as gels, while the TA10 probe has been considered the most suitable to analyze foam texture properties. These setups were selected to reflect how small variations in composition could impact the features of the final product [42].

The gel’s properties, such as firmness, consistency, adhesiveness, adhesive force, stringiness length, and stringiness work, were assessed using the TA-DEC probe and TA-BT-KIT fixture. The test parameters were the following: Test type—Compression, Pre-test speed—1.0 mm/s, Test speed—2.0 mm/s, Post-test speed—2.0 mm/s, Trigger load—10 g, Target value -10 mm. The results were recorded, and the texture parameters were calculated by using Texture ProCT Software 1.9 (Brookfield Engineering Laboratories, Middleboro, MA, USA). Firmness was calculated as the peak of the positive force (hardness) on the load vs. time graph, while consistency was determined as the area under the positive part of the graph (hardness work done). Adhesiveness refers to the work needed to overcome the attractive forces between the product’s surface (gel or foam) and the materials it comes in contact with. Adhesive force represents the force required to pull the probe from the product and it was measured as the total negative area and the adhesive force as the peak negative force. Stringiness length was calculated as the distance to which the gel extends before it breaks away from the probe while the stringiness work represents the work necessary to stretch the gel until the breaking point. The stringiness parameters give information about the gel behavior during stretching [42,43].

Foaming property (Foamability)

When talking about foaming cleansing products, the volume, stability, and density of the foam are important to consumers. Since most consumers correlate foamability with cleansing ability, they search for well-lathering formulations that generate stable and dense foam [30]. Foamability was assessed using the Cylinder shake method. A 50 mL sample from a 1% solution was placed in a 100 mL graduated cylinder (30 mm diameter × 250 mm height), inverted 20 times to generate foam, and the foam volume (mL) was measured. Three measurements were made for each formulation [30,44].

Foam stability

Foam stability was assessed by measuring the foam volume (mL) over time. Thus, the solutions used in the determination of foaming property were observed for 3 min, and the foam volume was recorded. Three experiments were conducted for each formulation and the mean value was recorded.

Dirt dispersion was evaluated by using the method previously described by AlQuadeib et al. [41] with small modifications. Briefly, 10 mL of distilled water, one drop of Indian ink, and one drop of cleansing product were added to a test tube and vigorously shaken 10 times. The amount of dye that passes into the foam immediately after shaking (the coloration of the foam) was rated on a scale from 1 to 4. To produce adequate cleansing products, it is beneficial to ensure that dirt remains in the water portion during the rinsing process. Lower values indicate better dirt dispersion, meaning the dirt remains in the water portion and is easier to rinse away.

pH measurement

The pH assessment was performed by using the pH meter (HI 8424, Hanna Instruments, Cluj-Napoca, Romania). The measurements were performed in triplicate, at 25 ± 0.2 °C.

Viscosity measurement

The viscosity of the optimal formulation at different shear rates was recorded using a rotational rheometer DV-III Ultra (Brookfield Engineering Laboratories, Middleborough, MA, USA, spindle 62). Measurements were performed in triplicate at 25 ± 0.2 °C and mean values ± standard deviation were reported.

#### 4.3.2. Characterization of the Foams

The foam was obtained through the Cylinder shake method from a 20 mL of 10% product solution placed in a 50 mL glass cylinder (20 mm diameter × 200 mm height), covered with a Parafilm^®^ type membrane, and shaken vigorously 20 times. The firmness, consistency, adhesiveness, adhesive force, stringiness length, and stringiness work done on the generated foam were determined using the Brookfield CT3 texture analyzer equipped with a TA10 probe. The recorded parameters were the following: Test type—Compression, Pre-test speed—1.0 mm/s, Test speed—2.0 mm/s, Post-test speed—2.0 mm/s, Trigger load—0.1 g, Target value—20 mm. All the determinations were performed in triplicate, and the mean value and standard deviation were reported.

Actuation force

The actuation force test was measured using a CT3 Texture Analyzer (Brookfield Engineering Laboratories, Middleboro, MA, USA) equipped with a TA10 probe. The recorded parameters were the following: Test type—Compression, Test speed—1.0 mm/s Trigger load—2 g, Target value—20 mm. The determinations were conducted in triplicate, in comparison with a commercial product.

The foam structure of the optimal formulation was determined using a Zeiss Primostar 3 Axiocam 208 color microscope (Zeiss, Jena, Germany) capturing at least three images of different samples of gel and foam with the Labscope application.

### 4.4. QbD Approach

#### 4.4.1. Definition of QTPP, CQA

Establishing the Quality Target Product Profile (QTTP), or the final objective of formulation development, is the first stage in the QbD process. The next action is to determine critical quality attributes (CQAs), critical material attributes (CMAs), and critical process parameters (CPPs), after which risk assessment is used to choose parameters that could have an impact on the quality of the final product [7].

#### 4.4.2. Risk Analysis

A Fishbone Diagram, also known as an Ishikawa or Cause-and-Effect Diagram, is a visual tool used to systematically identify the risks and the potential problems and root causes in a process. The factors noticed during the diagram development were then further analyzed and prioritized in a risk assessment [7].

Failure Modes and Effects Analysis (FMEA) is a systematic method for evaluating different processes to identify where and how they might fail and to assess the relative impact of the failures. The FMEA risk assessment involves calculating the Risk Priority Number (RPN) for each potential failure mode, which helps prioritize the risk factors. RPN is calculated by multiplying the values given for each parameter: severity (S), occurrence (O), and detection (D) of each factor. Each of them uses a scale from 1 to 10, where for S, 1 indicates insignificant impact while 10 indicates catastrophic impact on the user or process, respectively for O, 1 indicates extremely unlikely and 10 indicates very frequently. For D, 1 indicates a high likelihood of detection and 10 indicates a very low likelihood of detection [7].

#### 4.4.3. Experimental Design

Following the FMEA risk assessment, the variables with the greatest impact on the quality of the product were included as formulation variables in the experimental plan (concentrations of surfactants, polyols, and the gel-forming agent). The types and concentrations studied were established after multiple preliminary formulations to guarantee that the formulations subsequently generated by the experimental plan were organoleptically adequate and could be further analyzed. As response variables, both gel and foam properties were studied for a comprehensive understanding of how these factors influence the product, paving the way for future research and development in this field. Based on the formulation variables and responses presented in Table 5, a D-optimal experimental design with N = 13 experiments was generated (Table 6). Accordingly, the experiments were performed and the effects of independent variables on the responses were analyzed. Quality-of-fit assessment, coefficient calculation, and statistic parameters evaluation were performed with Modde 12.1 software (Sartorius, Germany).

#### 4.4.4. Statistical Analysis

The Modde 12 software was used to process the data from the experimental plan. The data was statistically analyzed by ANOVA test and a *p*-value below 0.05 was considered statistically significant.

## Figures and Tables

**Table 1:** QTPP and CQAs of foam dosage forms.

QTPP	Target	CQAs	Justification
Cosmetic dosage form	Cleansing foam	Yes	Surfactant-based product for dry skin cleansing
Application site	Face hygiene	-	
Product design	Foam	-	Impact on ease of use and spreading
Appearance	Smooth foam	-	Impact on users’ acceptability
Color	White	-	Impact on users’ acceptability
Odor	Specific for the herbal ingredients; fragrance-free	-	Impact on users’ acceptability
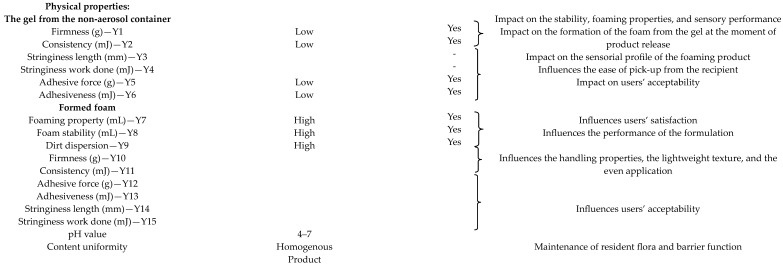			
Container	Foam container	-	Influences the formation of the foam/User- friendly delivery system

**Table 2:** The results of selected output responses.

Exp	Y1 (g)	Y2 (mJ)	Y3 (mm)	Y4 (mJ)	Y5 (g)	Y6 (mJ)	Y7 (mL)	Y8 (mL)	Y9	Y10 (g)	Y11 (mJ)	Y12 (g)	Y13 (mJ)	Y14 (mm)	Y15 (mJ)
N1	83.20 ± 9.80	13.52 ± 2.12	11.87 ± 0.33	5.51 ± 0.82	54.80 ± 6.40	8.44 ± 0.98	22.00 ± 0.57	20.00 ± 1.52	1.00 ± 0.57	6.80 ± 2.00	1.07 ± 0.46	6.70 ± 2.60	0.98 ± 0.43	34.21 ± 7.65	0.66± 0.33
N2	126.3 ± 3.80	22.77 ± 1.06	9.77 ± 0.39	8.05 ± 0.70	99.00 ± 3.60	14.95 ± 0.51	20.00 ± 0.57	19.00 ± 0.00	1.00 ± 0.57	6.30 ± 11.30	1.09 ± 0.47	3.80 ± 1.00	0.34 ± 0.27	37.04 ± 10.01	0.08± 0.27
N3	31.20± 4.00	4.12 ± 0.22	1.66 ± 0.74	0.08 ± 0.05	10.50 ± 3.00	0.64 ± 0.11	25.00 ± 0.57	23.00 ± 0.57	4.00 ± 0.57	6.30 ± 0.30	1.81 ± 0.22	3.50 ± 0.50	0.17 ± 0.11	56.27 ± 3.81	0.10± 0.16
N4	25.70 ± 2.00	4.30 ± 0.49	15.08 ± 20.00	0.22 ± 0.30	8.5 ± 0.00	0.66 ± 0.07	25.00 ± 2.00	23.00 ± 2.51	3.00 ± 0.57	8.20 ± 0.30	2.22 ± 0.3	3.20 ± 0.30	0.06 ± 0.05	56.94 ± 3.74	0.27± 0.07
N5	96.30 ± 6.50	17.47 ± 1.22	11.18 ± 0.95	6.21 ± 0.24	69.30 ± 6.10	11.21 ± 0.88	20.00 ± 2.00	19.00 ± 2.51	3.00 ± 0.57	5.70 ± 1.30	1.33 ± 0.35	5.00 ± 1.70	0.66 ± 0.37	52.79 ± 11.17	0.38± 0.08
N6	143.00 ± 1.80	26.12 ± 0.76	9.83 ± 0.73	9.08 ± 0.98	113.20 ± 3.20	16.88± 0.40	17.00 ± 0.57	18.00 ± 1.15	3.00 ± 1.00	6.50 ± 0.50	1.74 ± 0.26	3.30 ± 0.30	0.07 ± 0.08	50.05 ± 7.27	0.41± 0.17
N7	31.20 ± 4.90	4.13 ± 0.29	1.83 ± 0.59	0.10± 0.05	12.00 ± 3.50	0.83 ± 0.20	21.00 ± 1.52	19.00 ± 1.15	1.00 ± 0.57	5.20 ± 0.30	1.42 ± 0.19	5.00 ± 0.00	0.47 ± 0.14	58.71 ± 0.85	0.36± 0.20
N8	63.70 ± 6.40	9.11 ± 2.81	12.01 ± 8.11	2.96 ± 1.83	33.80 ± 2.30	4.66 ± 0.56	19.00 ± 1.15	18.00 ± 1.00	1.00 ± 0.00	9.00 ± 0.50	2.87± 0.26	3.30 ± 0.60	0.14 ± 0.09	57.74 ± 1.33	0.03± 0.15
N9	36.70 ± 5.30	3.61 ± 0.23	3.16 ± 2.28	0.21 ± 0.05	19.70 ± 2.80	0.77 ± 0.07	21.00 ± 1.00	20.00 ± 0.57	3.00 ± 0.57	6.20 ± 0.30	1.39 ± 0.35	5.00 ± 0.50	0.76 ± 0.26	58.46 ± 1.77	0.71± 0.28
N10	57.80 ± 6.90	7.55 ± 1.86	8.09 ± 5.08	1.72 ± 0.88	29.00 ± 4.30	3.96 ± 0.87	20.00 ± 0.57	20.00 ± 0.57	1.00 ± 0.00	5.20 ± 0.80	1.33 ± 0.18	4.30 ± 0.30	0.26 ± 0.08	56.74 ± 1.82	0.01± 0.13
N11	43.50 ± 2.00	7.96 ± 0.44	15.68 ± 0.97	2.10 ± 0.37	19.20 ± 1.00	3.28 ± 0.11	20.00 ± 0.00	19.00 ± 0.00	2.00 ± 0.57	5.70 ± 0.30	1.50 ± 0.07	3.50 ± 0.05	0.13 ± 0.03	53.42 ± 6.66	0.30± 0.04
N12	49.20 ± 3.50	9.00 ± 0.63	15.00 ± 1.07	2.56 ± 0.61	23.50 ± 3.30	3.78 ± 0.46	20.00± 0.00	20.00 ± 0.00	1.00 ± 0.57	6.70 ± 2.10	1.34 ± 0.21	4.70 ± 0.60	0.46± 0.11	57.37 ± 2.34	0.32± 0.15
N13	74.00 ± 5.20	13.30 ± 1.03	13.90 ± 1.12	5.34 ± 1.03	47.70 ± 4.50	7.50 ± 0.90	21.00 ± 0.57	21.00 ± 0.57	2.00 ± 0.00	4.30 ± 0.30	1.11 ± 0.12	4.80 ± 0.30	0.44 ± 0.06	54.09 ± 8.45	0.26 ± 0.19

Quantitative variables and their variation levels: X1—% xanthan gum, X2—% surfactants (lauryl glucoside, decyl glucoside. sodium cocoyl isethionate). X3—% polyols (glycerin:xylitol); Characterization of the gel: Y1—firmness (g), Y2—consistency (mJ), Y3—stringiness length (mm), Y4—stringiness work done (mJ), Y5—adhesive force (g), Y6—adhesiveness (mJ), Characterization of the foam: Y7—foaming property (mL), Y8—foam stability (mL), Y9—dirt dispersion, Y10—firmness (g), Y11-consistency (mJ), Y12—adhesive force (g), Y13—adhesiveness (mJ), Y14—stringiness length (mm), Y15—stringiness work done (mJ).

**Table 3:** Statistical parameters for ANOVA test and quality of fit.

Response	R^2^	Adjusted R^2^	Q^2^	*p*-Value Regression	*p*-Value Lack of Fit	Model Validity	Reproducibility
Y1	0.847	0.796	0.710	0.001	0.322	0.715	0.872
Y2	0.899	0.879	0.846	0.000	0.658	0.895	0.854
Y3	0.714	0.619	0.693	0.008	0.693	0.908	0.510
Y4	0.837	0.783	0.677	0.001	0.168	0.553	0.917
Y5	0.837	0.783	0.712	0.001	0.322	0.716	0.863
Y6	0.847	0.817	0.778	0.000	0.362	0.745	0.876
Y7	0.799	0.733	0.625	0.002	0.067	0.322	0.948
Y8	0.868	0.824	0.775	0.000	0.776	0.936	0.741
Y9	0.764	0.685	0.624	0.003	0.493	0.822	0.714
Y10	0.490	0.321	0.342	0.095	0.521	0.836	0.349
Y11	0.682	0.576	0.414	0.013	0.086	0.387	0.901
Y12	0.766	0.650	0.384	0.012	0.517	0.834	0.661
Y13	0.732	0.598	0.403	0.020	0.384	0.760	0.699
Y14	0.888	0.850	0.840	0.000	0.180	0.570	0.940
Y15	0.657	0.486	0.404	0.050	0.660	0.895	0.369

R^2^—the percentage of the variation of the response explained by the model; Q^2^—the percent of the variation of the response predicted by the model; Characterization of the gel: Y1—firmness (g), Y2—consistency (mJ), Y3—stringiness length (mm), Y4—stringiness work done (mJ), Y5—adhesive force (g), Y6—adhesiveness (mJ), Characterization of the foam: Y7—foaming property (mL), Y8—foam stability (mL), Y9—dirt dispersion, Y10— firmness (g), Y11—consistency (mJ), Y12—adhesive force (g), Y13—adhesiveness (mJ), Y14—stringiness length (mm), Y15—stringiness work done (mJ).

**Table 4:** Composition of the optimal formulation of the cleansing foam and the results of the analysis.

Ingredients (INCI)	Role	%	Characterization	Theoretical Values	Practical Values	Difference %
Lauryl Glucoside	Mild surfactant [6,10]	8.73	Firmness (g) (Y1)	23.50	20.30	13.61
Decyl Glucoside	Mild surfactant [6,10]	8.73				
Sodium Cocoyl Isethionate	Mild surfactant [6,12]	8.73	Consistency (mJ) (Y2)	3.70	3.15	14.86
Glycerin	Humectant [26,27]	1.80				
Xylitol	Humectant [26,27]	0.36	Adhesive force (g) (Y5)	5.80	5.10	12.06
Xanthan Gum	Thickening agent [29]	0.45				
Sodium PCA	Moisturizer [23,24]	1.00	Adhesiveness (mJ) (Y6)	1.00	0.86	14.00
*Aloe Vera* Oil	Moisturizer, wound healing [14,15,38]	2.00				
*Cedrus Atlantica* Bark Oil	Antimicrobial, antioxidant, anti-inflammatory [16,17]	0.10	Foaming property (mL) (Y7)	23.60	22.10	6.35
*Cupressus Sempervirens* Oil	Antimicrobial, antioxidant [20]	0.10				
PEG-40 Hydrogenated Castor Oil	Solubilizer for oils, emollient [39]	3.00	Foam stability (mL) (Y8)	22.50	19.80	12.00
*Copaifera Officinalis* Resin Oil	Antimicrobial, antioxidant, anti-inflammatory, wound healing [18]	0.10				
Benzyl Alcohol, Salicylic Acid, Glycerin, Sorbic Acid (Cosgard^®^)	Preservative [40]	1.00	Dirt dispersion (Y9)	2.40	2.10	12.50
Aqua	Solvent	qs ad 100				

**Table 5:** Input and output variables of the experimental plan.

Quantitative Variables and Their Variation Levels (%)				Response Variables	
X1—% xanthan gum	0.4	0.6	0.8	Gel Y1—firmness (g), Y2—consistency (mJ), Y3—stringiness length (mm), Y4—stringiness work done (mJ), Y5—adhesive force (g), Y6—adhesiveness (mJ)	Foam Y7—foaming property (mL), Y8—foam stability (mL), Y9—dirt dispersion, Y10— firmness (g), Y11—consistency (mJ), Y12—adhesive force (g), Y13— adhesiveness (mJ), Y14—stringiness length (mm), Y15—stringiness work done (mJ)
X2—% surfactants (lauryl glucoside: decyl glucoside: sodium cocoyl isethionate = 1:1:1)	15	22.5	30		
X3—% polyols (glycerin:xylitol = 5:1)	2	7	12		

**Table 6:** Experimental design matrix.

Experiment	X1	X2	X3
N1	0.4	15	2
N2	0.8	15	2
N3	0.4	30	2
N4	0.8	30	2
N5	0.4	15	12
N6	0.8	15	12
N7	0.4	30	12
N8	0.8	30	12
N9	0.4	30	7
N10	0.6	22.5	7
N11	0.6	22.5	7
N12	0.6	22.5	7
N13	0.6	22.5	7

Quantitative variables and their variation levels: X1—% xanthan gum, X2—% surfactants (lauryl glucoside, decyl glucoside, sodium cocoyl isethionate). X3—% polyols (glycerin:xylitol).

## Data Availability

The raw data supporting the conclusions of this article will be made available by the authors upon request.

## Supplementary Materials

The following supporting information can be downloaded at: https://www.mdpi.com/article/10.3390/gels10080484/s1, Figure. S1. Fishbone diagram of cleansing foams development; Figure S2. Contour plots. Table S1. FMEA analysis of cleansing foams development, Table S2. The revised quantitative factor effects and the associated p-values.
