# Supplementary material for: QbD Approach in Cosmetic Cleansers Research: The Development of a Moisturizing Cleansing Foam Focusing on Thickener, Surfactants, and Polyols Content
Source: Gels. 2024 Jul 23;10(8):484. doi: 10.3390/gels10080484 (PMC11354169; doi:10.3390/gels10080484)
Supplement: Supplementary file 1 [file gels-10-00484-s001.zip › gels-3083103-supplementary.pdf]

Supplementary Materials

QbD Approach in Cosmetic Cleansers Research: The Development of a Moisturizing Cleansing Foam Focusing on Thickener, Surfactants, and Polyols Content

Cătălina Bogdan[1], Diana Antonia Safta [1*], Sonia Meda Iurian[2], Dyana Roxana Petrușcă[1], Mirela-Liliana Moldovan[1*]

1 Department of Dermopharmacy and Cosmetics, Faculty of Pharmacy, "Iuliu Hațieganu" University of Medicine and Pharmacy, 12 I. Creangă St., 400010 Cluj-Napoca, Romania; catalina.bogdan@umfcluj.ro (C.B.); diana.an.safta@elearn.umfcluj.ro (D.A.S.); dyana.roxa.petrusca@elearn.umfcluj.ro (D.R.P.); mmoldovan@umfcluj.ro (M.L.M.)

2 Department of Pharmaceutical Technology and Biopharmacy, Faculty of Pharmacy, "Iuliu Hațieganu" University of Medicine and Pharmacy, 41 V. Babeș St., 400012 Cluj-Napoca, Romania; sonia.iurian@umfcluj.ro (S.I.)

* Correspondence: diana.an.safta@elearn.umfcluj.ro (D.A.S.); mmoldovan@umfcluj.ro (M.L.M.)

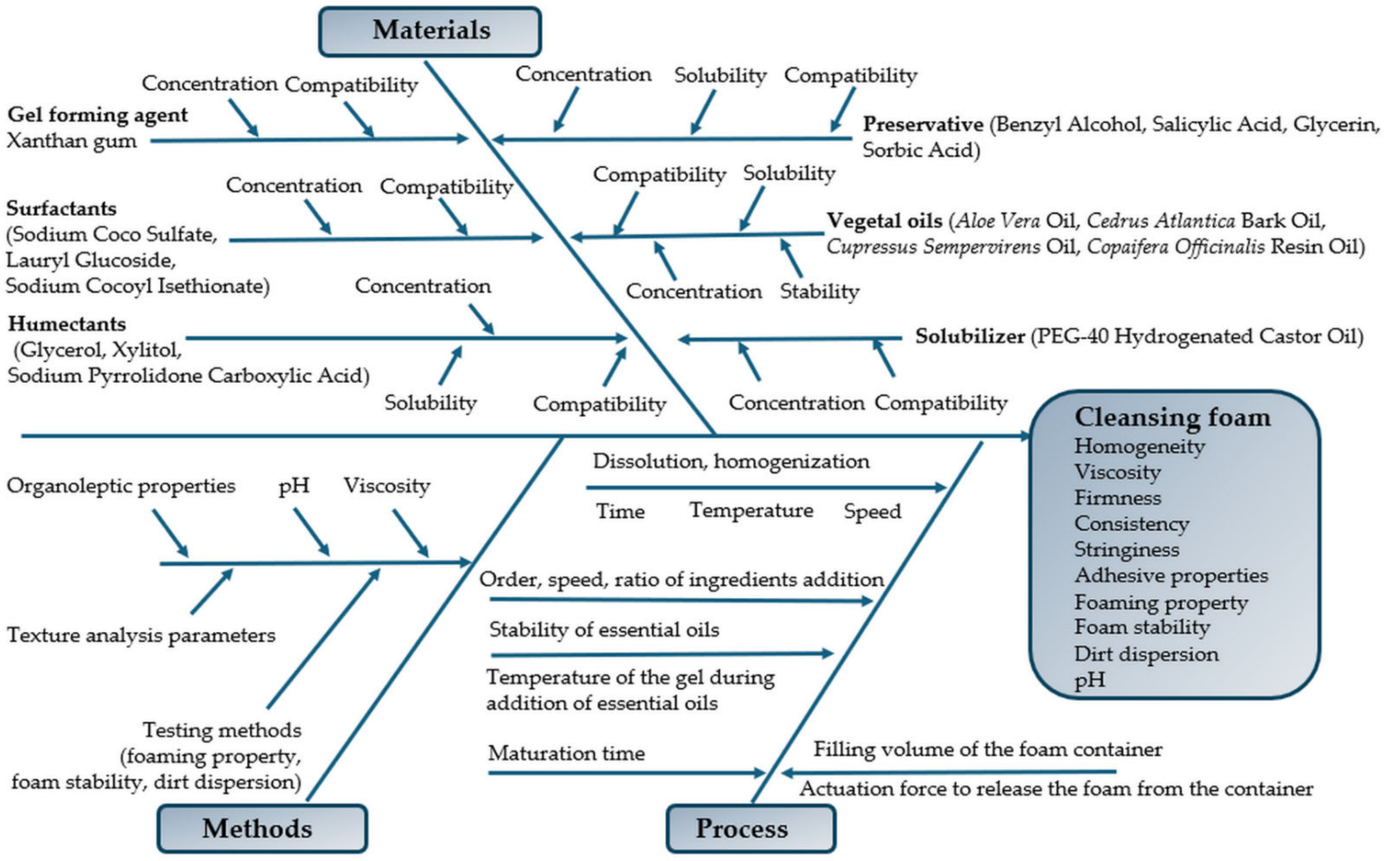

Figure S1. Fishbone diagram of cosmetic cleansing foams development.

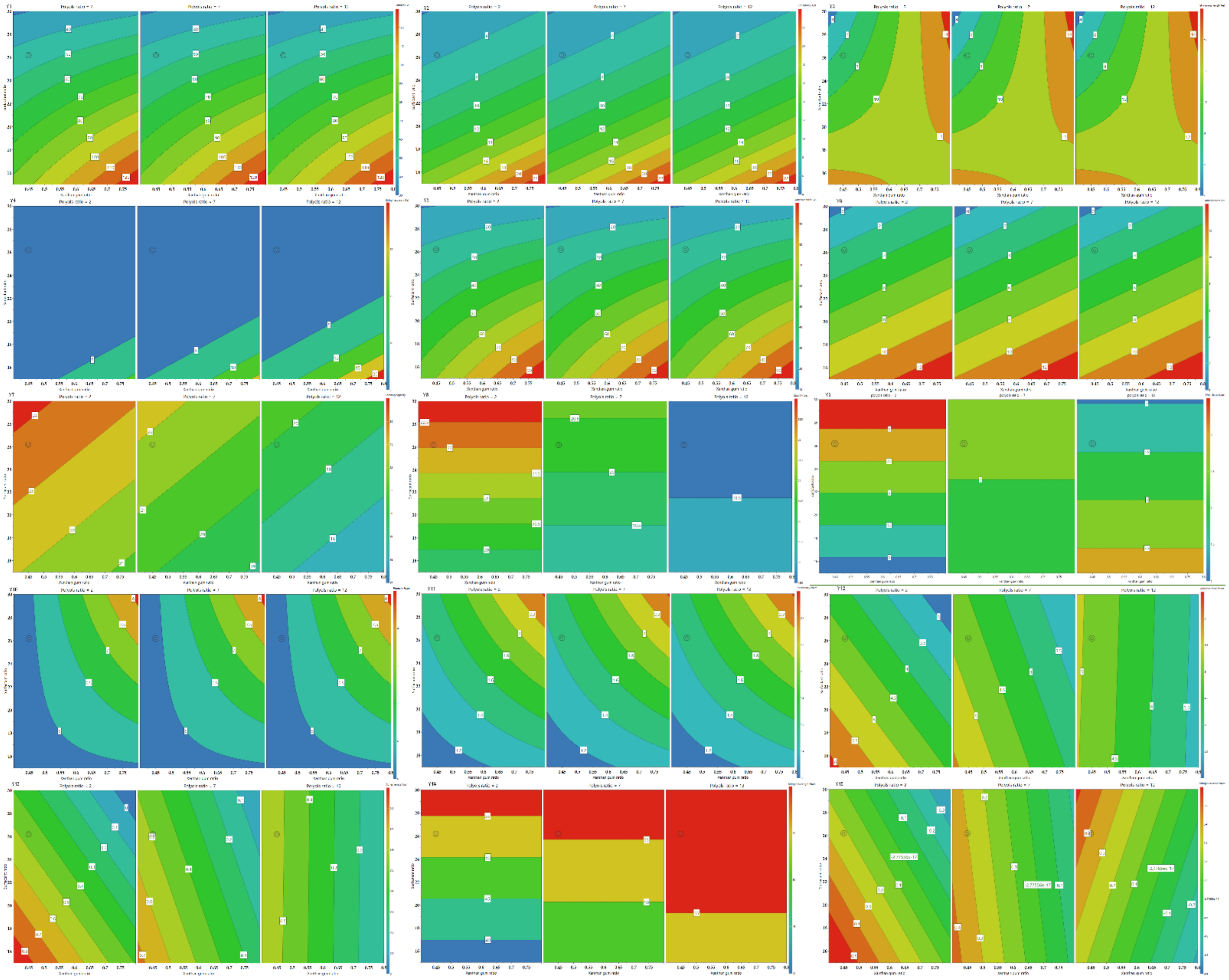

Figure S2. Contour plots.

Table S1. FMEA analysis of cosmetic cleansing foams development.

FMEA analysis for risk assessment								
**Factor**	**Failure mode**	**Failure effects**	**Potential causes**	**Control methods**	**O**	**S**	**D**	**RPN**
**Materials (CMAs)**								
Concentration of gel forming agent	Solubility issues and variations of product viscosity and texture parameters	Variable gel forming property, variable viscosity and texture parameters of the final product	Variation in concentration of gel forming agent	Establishment of an appropriate interval of concentrations to be studied by DoE, based on preliminary formulations	5	5	4	100
Concentration of surfactants	Solubility issues	Variable foam forming property, variable viscosity and texture parameters of the final product	Variation in concentration of surfactants	Establishment of an appropriate interval of concentrations to be studied by DoE, based on preliminary formulations	5	5	4	100

Concentration of humectants (polyols)	Solubility issues and variations of viscosity and texture parameters	Variable moisturizing capacity, variable viscosity and texture parameters of the final product	Variation in concentration of humectants (polyols)	Establishment of an appropriate interval of concentrations to be studied by DoE, based on preliminary formulations (the concentration of Sodium PCA was kept constant)	5	5	4	100
Stability of the vegetal oils	Stability issues of the vegetal oils	Variable quality and efficiency of the product	Degradation of the vegetal oils (oxidation etc.)	Addition of antioxidant (Benzyl Alcohol) and assuring controlled conditions during storage	2	5	2	20
Solubility of oils, concentration of solubilizer	Solubility issues	Non-homogeneity, variable viscosity and texture parameters	Variation in concentration of ingredients	Establishment of appropriate concentrations, based on preliminary formulations	2	5	1	10
Compatibility of ingredients	Incompatibility of ingredients	Non-homogeneity, instability	Interactions between chemical groups in structure of ingredients	Checking of the ingredients compatibility in specialty literature and based on preliminary formulations	2	5	1	10
			**Process (CPPs)**					
Homogenization time	Homogeneity issues	Non-homogeneity, variable quality and efficiency of the product	Variation in homogenization time, human error	Establishment of an appropriate homogenization time to guarantee the homogeneity of the products	3	4	1	12
Homogenization speed	Homogeneity issues	Non-homogeneity, variable quality and efficiency of the product	Variation in homogenization speed, human error	Establishment of an appropriate homogenization speed to be maintained constant, based on preliminary formulations	3	4	1	12
Homogenization temperature	Solubility issues Destructuration of gel, degradation of the vegetal oils	Non-homogeneity, variable quality and efficiency of the product	Degradation of the vegetal oils	Temperature monitoring, adding the vegetal oils after cooling the product	2	5	5	50
Order of adding the ingredients	Gel instability, homogeneity issues	Non-homogeneity, variable viscosity and texture parameters	Variation in order of adding the ingredients	Control of order of adding the ingredients	1	5	5	25
Filling volume of the foam container	Foam forming issues, foam instability	Variability of foam characteristics	Variation in filling volume (foam volume)	Control of the filling volume of the foam containers Verifying the fitting of the results by DoE	1	5	1	5
Actuation force to release the foam from the container	Foam forming issues, foam instability	Variability of foam characteristics	Variation in foam formation	Control of foam formation Verifying the fitting of the results by DoE	2	5	1	10

**Methods**								
Characterization methods	Non-reliable results	Variability of the results	Human and instrumental errors	Using previously validated methods, calibration and monitorization of the equipment<br>Verifying the fitting of the results by DoE	1	5	1	5

CMAs – critical materials attributes, CPPs – critical process attributes
O - occurrence, S - severity, D – detectability, RPN – risk priority number.

Table S2. The revised quantitative factor effects and the associated p-values.

	**Constant**	**X1**	**X2**	**X3**	**X1X2**	**X1X3**	**X2X3**
Firmness - Y1	65.65	11.19 (0.04)	-30.50 (0.00)	-	-6.69 (0.15)	-	-
Consistency - Y2	0.95	0.09 (0.02)	-0.25 (0.00)	-	-	-	-
Stringiness length - Y3	10.17	2.38 (0.02)	-1.52 (0.12)	-	2.51 (0.01)	-	-
Stringiness work done - Y4	0.163	0.24 (0.04)	-0.60 (0.00)	0.14 (0.21)	-	-	-
Adhesive force - Y5	40.85	9.91 (0.06)	-27.43 (0.00)	-	-7.41 (0.10)	-	-
Adhesiveness - Y6	5.97	1.57 (0.04)	-4.66 (0.00)	-	-	-	-
Foaming property - Y7	20.85	-0.59 (0.11)	1.01 (0.01)	-1.53 (0.00)	-	-	-
Foam stability - Y8	19.92	0.69 (0.01)	-1.18 (0.00)	-	-	-	-0.62 (0.01)
Dirt dispersion - Y9	2	0.19 (0.30)	*0.17 (0.35)	-	-	-	-0.79 (0.00)
Firmness - Y10	6.36	0.62 (0.07)	0.39 (0.22)	-	0.45 (0.12)	-	-
Consistency - Y11	1.57	0.27 (0.02)	0.31 (0.01)	-	0.14 (0.13)	-	-
Adhesive force - Y12	4.32	-0.74 (0.00)	-0.38 (0.06)	-0.03 (0.86)	-	-	0.34 (0.06)
Adhesiveness - Y13	0.38	-0.21 (0.00)	-0.10 (0.09)	-0.01 (0.80)	-	-	0.09 (0.09)
Stringiness length - Y14	52.60		5.94 (0.00)	3.33 (0.00)	-	-	-2.50 (0.01)
Stringiness work done - Y15	0.13	-0.25 (0.01)	-0.04 (0.59)	0.00 (0.95)	-	-	0.13 (0.07)

X1 - % xanthan gum, X2 - % surfactants, X3 - % polyols.
